# Is more always better? An S-shaped impact of gamification feature richness on exercise adherence intention

**DOI:** 10.3389/fpsyg.2025.1671543

**Published:** 2025-10-13

**Authors:** Yi Sun, Hongchi Dong, Wenyi Jiang

**Affiliations:** ^1^Xianda College of Economics and Humanities, Shanghai International Studies University, Shanghai, China; ^2^Shanghai Vocational College of Agriculture and Forestry, Shanghai, China

**Keywords:** gamification feature richness, digital exercise self-efficacy, exercise-adherence intention, S-shaped curve, self-determination theory (SDT)

## Abstract

Gamification is widely used to promote exercise adherence, yet “more features” do not always strengthen intention to sustain exercise plans. This study theorized and tested a curvilinear–specifically S-shaped–association between gamification feature richness and exercise adherence intention. Using polynomial modeling and slope analysis, we delineated engagement and overload zones across the observed feature-richness range. The results supported the S-shaped pattern: intention increased from low to moderate richness but weakened when feature sets became excessive. Digital exercise self-efficacy did not support the hypothesized inattention zone, but it amplified gains in the engagement zone and attenuated losses in the overload zone. These findings suggest that gamification yields diminishing and negative returns beyond a “right-sized” set of features, and that designers and practitioners should avoid feature bloat while providing adaptive controls that allow users to streamline secondary mechanics.

## Introduction

University campuses are commonly depicted as hubs of youthful vitality, yet epidemiological data reveal a stubbornly high prevalence of physical inactivity among tertiary students worldwide. In a pooled analysis of 23 countries, nearly two-thirds of 18–24-year-olds did not meet the WHO guideline of 150 min of weekly moderate-to-vigorous physical activity (MVPA) (Guthold et al., 2020), and activity patterns established in this developmental period tend to track into adulthood (Telama et al., 2014). This persistent shortfall motivates scalable, theory-informed approaches to strengthening exercise adherence in student populations.

Against this backdrop, mobile health (m-health) technologies are frequently trumpeted as scalable tools to promote exercise adherence (Direito et al., 2017; Domin et al., 2021). Adoption of fitness applications has expanded rapidly, particularly among Gen Z and university students. Meta-analytic evidence indicates that app-based interventions can yield small-to-medium improvements in weekly MVPA, especially when behavior change techniques such as goal-setting, feedback, and social comparison are embedded (Romeo et al., 2019). Gamification—the use of game-design elements in non-game contexts—now underpins many fitness apps (Johnson et al., 2016; Koivisto and Hamari, 2019; Lister et al., 2014), deploying leaderboards, badges, virtual challenges, narrative worlds, and adaptive “AI coaches” to enhance enjoyment and support autonomy, competence, and relatedness (Eppmann et al., 2018). While reviews consistently report positive average effects, they also note substantial heterogeneity, with some interventions attenuating or backfiring over time (Mazeas et al., 2022).

A key but under-studied driver is gamification feature richness—the breadth and density of gameful elements embedded in an app. Drawing on information-richness theory (Daft and Lengel, 1986) and recent human–computer interaction work (Roy et al., 2025), we define GFR as the perceived variety, multiplicity, and novelty of mechanics that a user can access during interaction with a fitness platform (Nacke and Deterding, 2017). Whereas most studies treat game elements as a binary treatment (present vs. absent) or count a handful of “core” mechanics, the burgeoning commercial marketplace evidences a combinatorial explosion of micro-features—daily streaks, surprise loot boxes (Johnson et al., 2016; Koivisto and Hamari, 2019), AI-generated workout “quests,” location-based augmented-reality races, and algorithmic social matchmaking, to name a few.

Intuitively, a richer set of features should provide more pathways to satisfy the basic psychological needs identified by self-determination theory (SDT; Deci and Ryan, 2000). Yet anecdotal user reviews and drop-off analytics from industry reports (A’Naja et al., 2024) hint that excessive complexity may overwhelm novices, dilute focal rewards, and ultimately reduce engagement—echoing classic findings that menu over-choice can depress satisfaction (Iyengar and Lepper, 2000). Conceptual models of “gamification fatigue” (Koivisto and Hamari, 2019) posit a non-linear trajectory: initial excitement, followed by satiation and, if stimuli escalate unabated, psychological fatigue. Prior work often operationalizes non-linearity with a quadratic term, which assumes a single optimum and treats “too little” and “too much” richness as symmetric around that point. This symmetry obscures the possibility that satiation (diminishing need support) and overload (need thwarting) are distinct processes that emerge at different thresholds. This study therefore theorizes and tests a cubic form that can identify two inflection points and three design regimes, allowing sharper, zone-specific predictions about when added features continue to scaffold motivation, when gains flatten, and when additional features become counterproductive.

According to social cognitive theory (Bandura, 1977), self-efficacy—one’s belief in the ability to execute a behavior under diverse circumstances—buffers against external barriers. Applied to the digital realm, digital exercise self-efficacy (DSE) reflects confidence in maintaining physical activity using predominantly app-based guidance (Resnick and Jenkins, 2000). Within an SDT account, we model digital exercise self-efficacy (DSE) as a moderator because it calibrates how the same gamified features are appraised—as need-supportive (informational, choice-enhancing) versus need-thwarting (controlling, cognitively taxing)—thereby altering both the strength and the shape of the GFR and EAI association. High-DSE users, whose experiences more readily satisfy the SDT need for competence, are likelier to interpret increasing feature richness as autonomy-consistent and competence-affirming, sustaining autonomous motivation across a wider range of richness; low-DSE users more readily experience the same cues as overwhelming or pressuring, hastening need thwarting and shifting the “sweet-spot/overload” thresholds leftward (Deci and Ryan, 2000; Ryan and Deci, 2017). Empirically, SDT syntheses show that need support versus need thwarting robustly differentiates motivational quality and adherence in exercise contexts, consistent with DSE functioning as a boundary condition rather than a conduit (Teixeira et al., 2012; Bartholomew et al., 2011; Ng et al., 2012).

Therefore, the present study advances the gamified-fitness literature by addressing three intertwined lacunae, each with a tailored contribution. First, although isolated field experiments hint that gamification effects are non-linear (Rapp et al., 2019), no study has formally modeled a full cubic S-curve or identified the “sweet-spot” at which benefits saturate and the “overload” point at which they reverse. We close this gap by applying higher-order polynomial regression and Johnson–Neyman breakpoint analysis to map the complete S-shaped trajectory linking GFR to exercise-adherence intention, thereby furnishing actionable thresholds for feature-release strategies. Second, while exercise self-efficacy is a robust direct predictor of adherence (McAuley et al., 2011), its capacity to buffer users from gamification overload remains untested, especially among digitally fluent yet physically inactive university students (Guthold et al., 2020). We position DSE as a moderating boundary condition and demonstrate that high-DSE students are insulated from the negative slope of the overload segment, whereas low-DSE peers are not, thereby extending social cognitive theory into the gamified m-health domain and offering segment-specific design guidance for campus wellness programmers. Collectively, these contributions furnish new measurement tools, rigorous non-linear modeling, and nuanced boundary-condition insights that together move the field beyond “more features are better” toward evidence-based optimization of gamified fitness platforms.

## Theory and hypothesis

### Self-determination theory (SDT)

Self-determination theory posits that sustained exercise adherence is largely contingent on the satisfaction of three fundamental psychological needs—autonomy, competence, and relatedness (Deci and Ryan, 2000). Within digital fitness platforms, these needs are addressed through gamification elements: autonomy is supported by personalized goal-setting, flexible challenge selection, and customized workout pathways; competence is enhanced through progressive feedback, adaptive difficulty levels, and visible achievements; relatedness is fostered by social interactions, leaderboards, and community-driven events (Eppmann et al., 2018). Thus, incremental increases in perceived GFR—the perceived variety, novelty, and density of available game-like functions—initially augment exercise motivation by providing multiple channels to fulfill psychological needs.

However, SDT also recognizes the possibility of need saturation and thwarting, which may arise when environments become overly controlling or cognitively demanding (Vansteenkiste and Ryan, 2013). Once the optimal threshold of feature richness is surpassed, further increases yield diminishing motivational returns and may even reverse the benefits due to cognitive overload. Excessive gamification features—manifesting as too many badges, constant pop-ups, frequent notifications, and excessively complex social comparisons—can shift the user experience from autonomy-supportive to autonomy-thwarting, undermining competence through informational overload, and impairing relatedness by diluting meaningful social interactions. Thus, beyond a critical tipping point, higher GFR may paradoxically lower adherence intentions, generating an overall cubic (S-shaped) trajectory.

Additionally, individual differences in users’ DSE, which are defined as one’s confidence in effectively utilizing digital exercise apps (Malodia et al., 2023; Ulfert-Blank and Schmidt, 2022), may moderate the overload segment of this relationship. High-DSE users possess stronger coping resources to navigate complex interfaces, buffering them against cognitive overload and preserving their autonomy and competence experiences (Maran et al., 2022). In contrast, low-DSE users likely interpret the same richness level as intimidating, amplifying perceived cognitive burden and hastening disengagement. Consequently, DSE emerges as a crucial boundary condition, determining how sharply adherence intentions decline once the optimal threshold of gamification feature richness is exceeded (Maran et al., 2022). This integrated SDT-based framework thus predicts an S-shaped relationship between GFR and adherence intention, moderated by digital exercise self-efficacy.

### GFR and exercise adherence intention

At the lower extreme of the richness continuum, a fitness app contains only rudimentary game artifacts—perhaps a single progress bar or a default badge. SDT argues that such sparse input leaves the need for autonomy, competence, and relatedness essentially “un-addressed” (Deci et al., 2017). Autonomy remains dormant because there are few meaningful choices; competence is neither challenged nor rewarded; relatedness is absent without social cues. In SDT terminology, the environment is non-controlling but also non-supportive, yielding amotivation rather than active engagement.

Extant m-Health research corroborates this “insufficient dose” problem. Perski et al. (2017) summarized 85 digital-behavior-change interventions and found that apps providing fewer than three interactive features produced no measurable gains in weekly MVPA. From a cognitive-attention perspective, too little salience fails to cross the orienting threshold that triggers deeper processing (Lang, 2000). In other words, when GFR is very low, students neither feel stimulated nor overwhelmed; they simply fail to notice the sparse game cues embedded in the app. Under such conditions, behavioral intentions are driven by pre-existing habits or external constraints (e.g., mandatory physical-education credits), not by the app’s gamified scaffolding.

*H1a: GFR is unrelated to exercise adherence intention when the individual perceived GFR is at a low level.*

As GFR climbs into a moderate band, students encounter a diverse yet digestible array of game mechanics: an adaptive level system conveys progress, a weekly leaderboard sparks friendly rivalry, and an AI coach curates quests that align with personal goals. SDT predicts that such an environment maximally satisfies autonomy, competence, and relatedness. Autonomy is nurtured through choice; competence through calibrated feedback; relatedness through social comparison and cooperative challenges (Deci and Ryan, 2000). Empirical evidence underscores this motivational gain. Eppmann et al. (2018) validation of the GAMEX scale demonstrated that a moderate density of mechanics increased “gameful experience” scores and—critically—translated into higher self-reported workout frequency one month later (β = 0.42, *p* < 0.001). A meta-analysis conducted by Yang et al. (2022) positioned participants in low-, medium-, and high-feature versions of the same app; the medium-feature group logged 38 % more MVPA minutes than the low-feature group and outperformed the high-feature group by 19 %. Neuro-imaging studies also reveal heightened ventral-striatum activation—an index of intrinsic rewards—when users interact with moderately rich gamified dashboards (Lorenz et al., 2015; Stein et al., 2025). Cognitively, moderate richness sits below overload thresholds, allowing users to chunk information into coherent mental models (Sweller, 1988). The interactive variety sustains curiosity (Berlyne, 1960) and elicits “flow” states conducive to persistence (Csikszentmihalyi and Csikzentmihaly, 1990).

*H1b: GFR is positively related to exercise adherence intention when perceived GFR is at a moderate level.*

Beyond the optimal point, successive feature additions no longer expand motivational affordances; instead, they tax cognitive resources and may even thwart SDT needs. Overly frequent pop-up quests or social-feed alerts risk being perceived as controlling, thereby undermining autonomy; noisy, overlapping feedback messages can erode competence by obscuring clear performance signals. Social comparison features may morph from supportive to anxiety-inducing as leaderboard gaps widen, jeopardizing relatedness (Vansteenkiste and Ryan, 2013).

Empirical studies document this downturn. Koivisto and Hamari’s (2019) two-wave survey of 1,188 fitness-app users revealed that badge complexity was positively linked to “gamification burnout,” which in turn predicted app abandonment. In a qualitative study, Bieser and Hilty (2020) observed that individuals should put more energy into adopting complex technology. Cognitive-load experiments show that multi-panel dashboards elicit higher scores and poorer recall of workout goals (Nimbarte et al., 2024). From an SDT lens, such over-featured systems thwart needs by becoming controlling (too many notifications dictating behavior) or incoherent (difficulty discerning true mastery). Users experience reactance, fatigue, and disengagement—manifested behaviorally as declining adherence intentions.

*H1c. GFR is negatively related to exercise adherence intention when perceived GFR is at a high level.*

### The moderating role of DSE

Self-efficacy theory maintains that people’s beliefs in their capabilities determine how much stress or motivation they experience when facing environmental demands (Bandura, 1997). In a mobile-fitness context, these beliefs translate into DSE—confidence in one’s ability to locate, interpret, and exploit the ever-expanding repertoire of app functions to maintain regular exercise (Resnick and Jenkins, 2000). DSE is distinct from general computer self-efficacy because it is anchored in physical-activity goals; it is also distinct from traditional exercise self-efficacy because it presumes the digital mediation of action plans (Compeau and Higgins, 1995; Resnick and Jenkins, 2000). Below, we articulate how DSE interacts with gamification feature richness in each of the three motivational zones introduced earlier: inattention, engagement, and overload.

When perceived GFR lies at the lower extreme, an app offers so few game elements that it fails to register as a motivational agent. SDT calls this a “need-flat” environment: it neither supports nor thwarts autonomy, competence, or relatedness (Deci and Ryan, 2000). Under such sparse conditions, perhaps a lone progress bar or a generic step counter, students’ behavioral intentions are primarily driven by habitual routines or extrinsic obligations (e.g., compulsory physical-education credit), not by digital gamification cues. From a SET perspective, DSE can only operate on perceived challenges. Bandura (1997) argues that efficacy beliefs modulate effort “when people have some control over events that affect them.” Here, with virtually no game mechanics to master, both high- and low-DSE students confront an identical and trivially simple interface; there is nothing to control, personalize, or reinterpret. Thus, DSE cannot exert leverage; its variance remains dormant. Therefore, we propose the following hypothesis.

*H2a: Within the low level of GFR, digital exercise self-efficacy will not moderate the association between GFR and exercise adherence intention; specifically, GFR is unrelated to exercise adherence intention when GFR is at a low level, regardless of whether the DSE is high or low.*

As GFR rises into the engagement band, the platform furnishes a “Goldilocks” mixture of mechanics: adaptive level systems, AI-curated quests, social leagues, and time-limited challenges. These features collectively satisfy SDT needs—provided that users feel competent to navigate them. High-DSE students perceive each new mechanic as an opportunity to express volitional choice (autonomy), demonstrate skill (competence), and connect socially (relatedness). SET research shows that efficacy heightens attention to instrumentally relevant cues and increases exploratory usage (Compeau and Higgins, 1995). These behavioral patterns amplify the motivational returns that SDT predicts in the moderate richness range. Conversely, low-DSE students view the same array of features with partial uncertainty. They may exploit some mechanics (e.g., automatic badge accrual) yet ignore others requiring configuration (e.g., team challenges). The motivational yield of each incremental feature is therefore diluted. Therefore, we propose the following hypothesis.

*H2b: At the moderate level of GFR, the positive slope of GFR on exercise adherence intention will be steeper for students with higher DSE than for those with lower DSE.*

Beyond the optimal threshold, feature richness crosses into overload. Notifications multiply; multiple leaderboards display conflicting rankings; AI-generated daily quests stack atop weekly “boss battles.” Under such density, information-load theory predicts cognitive strain (Sweller, 1988), while SDT warns of need thwarting as prompts become controlling and competence feedback turns chaotic (Vansteenkiste and Ryan, 2013). The net effect is a downturn in adherence intention.

High-DSE students, however, bring metacognitive skills and confidence that allow them to filter, priorities, or deactivate non-essential features. Qualitative interviews by Janssen et al. (2024) reveal that high-efficacy users actively curtail app notifications, consolidate social feeds, and reinterpret ranking disparities as “data noise,” thereby preserving their sense of autonomy and competence. SET offers two mechanisms for this buffering. First, cognitive reappraisal: high-DSE individuals frame difficult tasks as surmountable; thus, an imposing interface becomes a solvable puzzle rather than a threatening barrier. Second, self-regulatory skills: high-DSE users are more likely to deploy time-management and self-instruction strategies, countering the attentional drain of feature overload.

*H2c: At the high level of GFR, the negative slope of GFR on exercise adherence intention will be attenuated (less steep) for students with higher DSE compared to those with lower DSE.*

## Materials and methods

### Sample and procedure

The study was conducted at a university in the Yangtze River Delta. A de-identified roster of all daytime students was obtained from the Registrar’s Office. To capture disciplinary breadth, faculties were first stratified into Humanities, Natural Sciences, Engineering, and Business. Within each faculty, one compulsory lecture course from every academic year (Years 1–4) was randomly selected.

Because the constructs under examination require lived experience with mobile fitness platforms, only students who had opened any exercise or step-tracking app at least once in the past 30 days were eligible (e.g., Keep, Huawei Health). This criterion was communicated in all recruitment materials and verified by the first survey item. In April 2025, instructors posted a standardized announcement on the university’s learning-management system (LMS) and mentioned it briefly in class. The notice invited recent app users to a “10-min questionnaire on digital exercise experiences,” outlining anonymity, voluntary participation, and a personalized feedback report as an incentive. Two automated LMS reminders were sent on Day 3 and Day 7 to students who had not yet clicked either the Yes (eligible) or No (ineligible) link.

Eligible students who clicked “Yes” were routed to a mobile-optimized online questionnaire that opened with a confirmation of recent app use and then flowed—without page breaks—through all study measures: demographics (gender, year code, height and weight for BMI, typical weekly app-use minutes), the 24 random-ordered Gamification Feature Richness items, a mid-list attention check (“Please select “4” for this statement”), the nine Digital Exercise Self-Efficacy items (also randomized) with a second attention check after item 5, and finally the three Exercise-Adherence-Intention items.

Of 657 students who began the survey, 643 satisfied the app-use filter. Eleven records were discarded: five failed both attention checks, three reported implausible BMI (< 15 kg/m^2^), and three were duplicate IP addresses (earlier timestamp retained). The resulting analytic sample numbered 632 students (54 % female; years, SD = 1.4; BMI M = 22.4, SD = 3.1). Item-level missingness averaged 0.4 % and was imputed via expectation–maximization. Mahalanobis-distance diagnostics flagged no multivariate outliers at *p* < 0.001.

### Measurement

#### Gamification feature richness

Guided by recent gamification reviews and scale-development work (Eppmann et al., 2018; Högberg et al., 2019; Koivisto and Hamari, 2019; Zainuddin et al., 2024), we identified six, literature-anchored dimensions of Gamification Feature Richness. (1) Levels and Badges capture hierarchical rewards cues that visualize progressive mastery and have been shown to heighten perceived competence and enjoyment. (2) AI motion-recognition reedback refers to a real-time, sensor-based technique correction—for example, form-checking via smartphone camera—that boosts competence beliefs in mobile-fitness contexts. (3) AI coach personalization denotes algorithm-generated workout plans that adapt to a user’s performance history, echoing the “guidance” facet of GAMEFULQUEST. (4) Dynamic challenges are novelty-rich, time-limited quests (e.g., weekend step streaks) that sustain engagement by periodically resetting goals. (5) Social competition; leaderboards comprise rankings, duels, and team races that leverage peer comparison to satisfy relatedness needs and predict higher workout frequency. (6) Virtual companion interaction involves AI avatars or chatbots that deliver encouragement and accountability prompts, a mechanism linked to increased adherence in avatar-mediated training studies. Three sport-technology scholars independently mapped candidate items to these dimensions (content-match ≥ 92 %); items with < 80 % agreement were rewritten or dropped. Each dimension is represented by four first-person statements (24 items in total).

Illustrative items include: “This app offers a wide range of levels and badges that I can progressively unlock” (Levels and Badges); “During workouts, the app detects my movements and instantly tells me how to improve my form” (AI Motion-Recognition Feedback); “The AI coach creates training plans that adapt to my goals and recent performance” (AI Coach Personalization); “I frequently receive new time-limited challenges that keep the workouts fresh” (Dynamic Challenges); “The leaderboard lets me compare my results with classmates or friends in real time” (Social Competition); and “A virtual trainer or avatar talks to me during sessions and encourages me to continue” (Virtual Companion Interaction). All items were rated on a seven-point agreement scale (1 = strongly disagree, 7 = strongly agree).

#### Digital exercise self-efficacy

Digital exercise self-efficacy was measured with a nine-item scale adapted from the Self-Efficacy for Exercise (SEE-9) instrument developed by Resnick and Jenkins (2000). To anchor confidence specifically in a mobile-fitness context, each item was prefixed with the phrase “Using only this fitness app…”. A representative item reads, “Using only this app, I could exercise even when I feel tired,” while others probe confidence under bad weather, academic workload, or lack of equipment. All items employed a seven-point confidence metric (1 = not confident at all, 7 = very confident).

#### Exercise adherence intention

Exercise adherence intention was assessed with a concise three-item scale adapted from Ahn et al. (2016), which taps students’ prospective commitment to maintain regular physical activity. Each statement explicitly referenced the focal fitness app to ensure contextual alignment, for example: “I intend to keep exercising regularly with this app over the next month.” Respondents rated their agreement on the same seven-point Likert continuum used elsewhere (1 = strongly disagree, 7 = strongly agree).

### Control variables

Three background factors were entered as covariates because prior research links each to exercise motivation or to the way students respond to digital cues. First, gender (0 = female, 1 = male) was controlled because men and women differ both in technology-adoption patterns (Venkatesh and Morris, 2000) and in leisure-time physical-activity prevalence (Trost et al., 2002). Second, body-mass index (BMI) can shape both exercise self-efficacy and responsiveness to feedback—higher BMI is associated with diminished confidence in completing workouts (McAuley et al., 2011)—so BMI (kg m^2^) was grand-mean centered and entered. Finally, academic year (dummy-coded 1 = freshman through 4 = senior) captured curricular workload differences; longitudinal evidence shows MVPA tends to decline after the first university year (Bray and Born, 2004). Controlling for these four variables helps ensure that any S-shaped effect of Gamification Feature Richness and any buffering by Digital Exercise Self-Efficacy are not artifacts of demographic or corporeal confounds.

## Results

### Reliability

All three study instruments demonstrated excellent reliability. The 24-item GFR scale returned a Cronbach’s α of 0.98, indicating an exceptionally high degree of inter-item homogeneity and confirming that the six sub-dimensions cohere around a single higher-order construct. The nine-item DSE scale achieved an α of 0.95, likewise signifying that its confidence statements operate as a tightly integrated set. Finally, even the brief three-item Exercise-Adherence Intention scale produced an α of 0.94, well above the.70 threshold commonly recommended for research instruments (Nunnally and Bernstein, 1994). These coefficients suggest minimal measurement error and provide a solid psychometric foundation for the subsequent hypothesis tests.

### Validity

We conduct confirmatory factor analysis in AMOS 23.0. Key goodness-of-fit statistics indicate that the confirmatory factor model is highly satisfactory. First, the χ^2^/df ratio is 1.03, well below the 3.0 cut-off normally used to flag misfit. Second, the Comparative Fit Index reaches.99 and the Tucker-Lewis Index.99, both above the.90 benchmark, showing the specified structure explains the data almost perfectly relative to an independence model. Third, the Standardized Root Mean Square Residual (SRMR) is.02, comfortably under the stringent.05 threshold and signaling minimal average residual error. Finally, an absolute fit indicator, the Goodness-of-Fit Index, stands at.95, again exceeding the.90 criterion. Collectively, the latent variables are well represented by their items and the overall measurement model is a good reflection of the observed covariance pattern. Therefore, the construct validity is acceptable for this study.

### Correlations

The correlations are presented in Table 1. The results showed that students who perceive their fitness app as more gamified report a moderate increase in exercise-adherence intention (r = 0.41, *p* < 0.001), while Gamification Feature Richness shows a small but significant link with digital exercise self-efficacy (r = 0.20, *p* < 0.001). In turn, higher self-efficacy is also weakly related to stronger adherence intention (r = 0.19, *p* < 0.001). The pattern suggests that richer game elements are associated with both greater confidence in using the app and a stronger commitment to keep exercising, yet the modest magnitudes leave ample room for the non-linear and moderating effects tested in the subsequent analyses.

**TABLE 1:** Mean, standard deviations, and correlations.

Variables	Mean	S.D.	1	2	3	4	5	6
1. Gender	0.47	0.50						
2. Year	2.41	1.11	0.02					
3. BMI	22.30	2.98	−0.06	−0.03				
4. GFR	4.04	1.55	−0.04	0.05	0.00	(0.98)		
5. DSE	5.10	1.10	0.01	0.02	0.00	0.20^***^	(0.95)	
6. EAI	5.37	1.11	0.05	0.04	0.05	0.41^***^	0.19^***^	(0.94)

N = 632;

^***^p < 0.001; GFR, gamification feature richness; DSE, digital exercise self-efficacy; EAI, exercise-adherence intention; The value in the parentheses on the diagonal are the Cronbach’s alpha coefficients.

### Hypothesis testing

We conduct regression analysis in STATA 16.0 to examine the hypotheses; the results are presented in Table 2. Model 1, containing only the four demographic controls, explained a negligible share of variance in exercise-adherence intention. Adding the linear Gamification amification near Gamificy the four demograpsubstantial increment; the positive coefficient (β = 0.30, *p* < 0.001) indicates that, on average, richer gamification is associated with higher adherence intent. Model 3 introduced the quadratic component (GFR^2^), which proved negative and significant (β = −0.29, *p* < 0.001), signaling a decelerating trend. Model 4 added the cubic term (GFR^3^), also negative (β = −0.11, *p* < 0.001). Collectively, the pattern β_1_ > 0, β_2_ < 0, β_3_ < 0 satisfies the formal criteria for a sigmoidal (S-shaped) curve, which provides the initial evidence for supporting Hypothesis 1a–1c.

**TABLE 2:** Regression results.

Variables	Exercise-adherence intention					
	Model 1	Model 2	Model 3	Model 4	Model 5	Model 6
Gender	0.11	0.15	0.08	0.09	0.09	0.07
Age	−0.02	−0.03	−0.03	−0.03	−0.02	−0.02
Year	0.04	0.02	0.05	0.05	0.04	0.05
BMI	0.02	0.02	0.02	0.02	0.02	0.02
GFR		0.30^***^	0.30^***^	0.82^***^	0.80^***^	0.79^***^
GFR^2^			−0.29^***^	−0.31^***^	−0.31^***^	−0.32^***^
GFR^3^				−0.11^***^	−0.11^***^	−0.10^***^
DSE					0.12^***^	0.14^***^
GFR^*^DSE						−0.06
GFR^2^^*^DSE						−0.01
GFR^3^^*^DSE						0.03^**^
R^2^	0.01	0.18^***^	0.57^***^	0.66***	0.67^***^	0.69^***^
ΔR^2^						0.02^*^

N = 632, the coefficients are unstandardized;

*p < 0.05,

^**^p < 0.01,

^***^p < 0.001; GFR, gamification feature richness; DSE, digital exercise self-efficacy, EAI, exercise-adherence intention.

**FIGURE 1:**
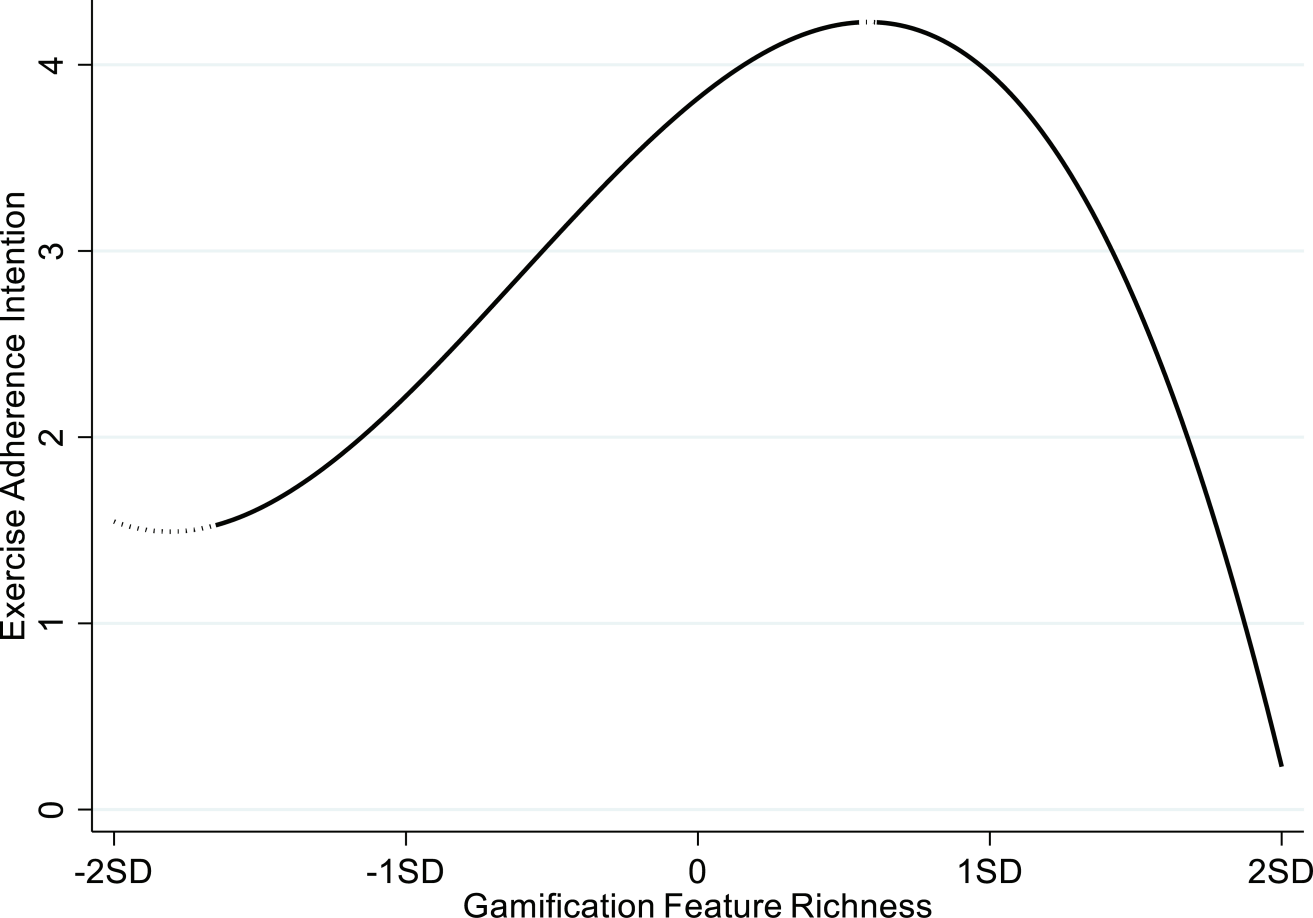
The S-shaped relationship between gamification feature richness and exercise adherence intention.

Additionally, we conduct simple slope tests proposed by Aiken and West (1991) to further test Hypothesis 1a–1c. Table 3 reports conditional slopes of GFR across its distribution. At very low richness (−2 SD), the slope is negative but not significant (t = −1.80). The first statistical “turn-on” point emerges at GFR = −2.58, after which slopes become significantly positive and peak around the mean (0 SD, slope = 0.82, t = 18.78). A second threshold appears near.85; beyond.95, the slope flips negative and grows rapidly steeper (e.g., slope = −0.90 at +1 SD). Collectively, for very low Gamification amification, for very < −2.58), the slope is statistically nil; therefore, H1a is supported. When GFR is located between −2.58 and 0.85, it turns sharply positive; therefore, H1b is supported; when GRF beyond 0.95, the slope flips negative and grows steeply more so, therefore, H1c is supported. Figure 1 depicts the S-shaped relationship between GFR and exercise adherence intention.

**TABLE 3:** Simple slope tests for S-shape effect.

GFR	Slopes	T value
−2 SD	−0.37	−1.80
Threshold 1 (−2.58)	0.26^*^	1.99
−1 SD	0.99^***^	24.74
0	0.82^***^	18.78
Threshold 2 (0.85)	0.07^*^	2.07
Threshold 3 (0.95)	−0.07^*^	−2.16
1 SD	−0.90^***^	−20.70
2 SD	−4.15^***^	−19.17

N = 632,

*p < 0.05,

^***^p < 0.001; GFR, gamification feature richness.

Model 5 added DSE as a covariate (β = 0.12, *p* < 0.001). Finally, Model 6 entered the three interaction terms; only the cubic interaction (GFR^3^ × DSE) was significant (i = 0.03, *p* < 0.01), nudging R^2^ to 0.69 and contributing an additional 2 % variance—evidence that DSE conditions the overload segment of the curve, which provides initial evidence for supported Hypothesis 2a-2c.

Simple slope tests were run separately for students one standard deviation below (low DSE) and above (high DSE) the mean (see Table 4). When digital self-efficacy is low, Gamification Feature Richness (GFR) reduces exercise-adherence intention in the very sparse range: between −2 SD and −2.76, the slope is significantly negative (−0.97, *p* < 0.001). This detrimental influence disappears once richness exceeds −2.76, and in the band from –2.76 up to –2.28, the effect is statistically neutral. Crossing –2.28 marks a turning point: GFR now exerts a significant positive impact that accelerates through the engagement zone, peaking near –1 SD (slope = 0.86, *p* < 0.001) to the mean (slope = 0.89, *p* < 0.001). Yet the benefit is short-lived; after 0.81, the slope dwindles to a marginal 0.09 and becomes non-significant by 0.94. Once GFR surpasses 0.94, the curve turns downward again and the influence becomes significantly negative (e.g., −1.07 at + 1 SD, *p* < 0.001; −4.89 at + 2 SD, *p* < 0.001), steepening into the overload region.

**TABLE 4:** The simple slope tests for moderating effect.

Variable	GFR	Slopes	T value
Low DSE	−2 SD	−0.97	−3.64^***^
	Threshold 1 (−2.76)	−0.34	−1.97^*^
	Threshold 2 (−2.28)	0.26	2.00^*^
	−1 SD	0.89	16.69^***^
	0	0.86	14.37^**^
	Threshold 3 (0.81)	0.09	2.01^*^
	Threshold 4 (0.94)	−0.08	2.01^*^
	1 SD	−1.07	−16.41^***^
	2 SD	−4.89	−15.84^***^
High DSE	−2 SD	0.55	1.77
	Threshold 1 (−3.04)	0.59	2.00^*^
	−1 SD	1.18	16.90^***^
	0	0.72	12.13^***^
	Threshold 2 (0.76)	0.09	2.02^*^
	Threshold 3 (0.93)	−0.09	2.16^*^
	1 SD	−0.84	−17.17^***^
	2 SD	−3.50	−12.41^***^

N = 632,

*p < 0.05,

^**^p < 0.01,

^***^p < 0.001; GFR, gamification feature richness; DSE, digital exercise self-efficacy.

**FIGURE 2:**
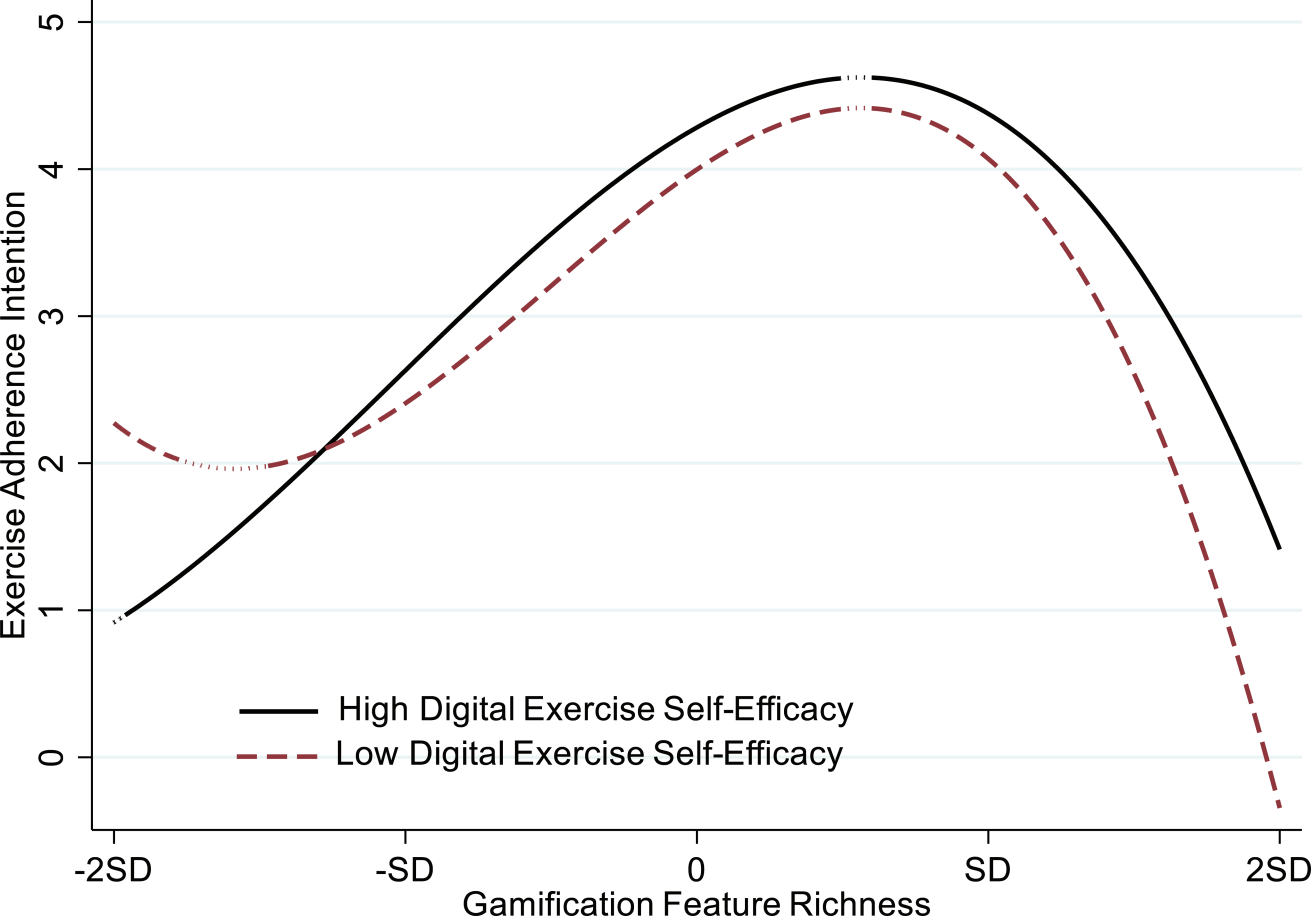
The moderating effect of digital exercise self-efficacy on the S-shaped relationship between gamification feature richness and exercise adherence intention.

With high digital self-efficacy, the landscape shifts. In the lowest segment (−2 SD to −3.04), the positive slope is small and not significant, indicating that very sparse gamification neither hinders nor helps. Beyond −3.04, GFR begins to aid adherence intentions, and the positive effect intensifies across the moderate-richness band, reaching 1.18 at −1 SD (*p* < 0.001). The advantage then tapers: at 0.76, the slope drops to 0.09 (*p* < 0.05) and loses significance shortly after. A reversal occurs only when richness passes 0.93, where the slope turns significantly negative, though the drop (−0.84 at + 1 SD) is gentler than that experienced by the low-efficacy group. Figure 2 depicts the moderating effect of DES on the S-shaped relationship between GFR and exercise adherence intention. In sum, high-DSE students start benefiting earlier, achieve a higher peak, and encounter a milder decline once feature richness becomes excessive, illustrating the buffering power of self-efficacy predicted by the moderation hypotheses.

First, as predicted, the GFR × DSE interaction was statistically non-significant across most of the sparse-feature band, and high-DSE students showed no reliable change in adherence intent—evidence that efficacy remains largely inert when gamified cues are minimal. However, low-DSE students experienced a small but significant negative slope in the extreme left tail (−2 SD to −2.76), indicating that very sparse gamification can actually undermine adherence among those who feel least confident with the app. Because this detrimental effect was confined to the far edge of the benefited from added features, yet the positive distribution, H2a is only partially supported. Second, both groups slope was markedly steeper for high-DSE students (1.18 at the mean) than for low-DSE peers (0.86), and the cubic interaction term was significant (β = 0.03, *p* = 0.021), thus H2b is supported. Third, once richness became excessive, the GFR–adherence slope turned negative for all respondents, but the decline was milder among high-DSE students (−0.84 at + 1 SD) than among low-DSE students (−1.07 at + 1 SD). This attenuated downturn confirms the buffering role proposed in H2c, which is therefore supported.

These findings establish the statistical pattern; in the next section, we discuss their theoretical and practical implications.

## Conclusion and discussions

### Theoretical implications

Whereas prior work has typically modeled feature richness with a quadratic (inverted-U) term implying one turning point, our cubic specification reveals two inflection points that segment three design regimes. At low-to-moderate richness, additional features can scaffold autonomy, competence, and relatedness (Manninen et al., 2022); beyond the first inflection point, marginal gains flatten; and after the second inflection point, added features increasingly risk informational overload or controlling cues that thwart needs (Bartholomew et al., 2011; Howard et al., 2025). This multi-regime view yields more precise, zone-specific prescriptions and helps reconcile mixed findings, because the location of the inflection points shifts with digital exercise self-efficacy, effectively widening the viable design space at higher digital exercise self-efficacy levels (e.g., Encantado et al., 2023; Morbée et al., 2024).

A first theoretical insight is the way our cubic pattern bridges the motivational lens of SDT with the cognitive-load warnings of information-overload research. Classic SDT experiments have repeatedly shown that adding badges, levels, or social quests can heighten need satisfaction and increase exercise engagement (Eppmann et al., 2018), yet field data on commercial apps often document rapid “feature fatigue” and churn once interfaces become cluttered (Koivisto and Hamari, 2019). By estimating a cubic rather than quadratic function, we reveal why both findings can be simultaneously true: SDT-consistent gains dominate up to a data-driven sweet-spot, after which an overload tipping-point appears where cognitive cost and autonomy-thwarting notifications outweigh competence feedback—echoing overload theorists’ claim that excessive informational variety becomes a liability (Mengis and Eppler, 2004). Our third-order term explicitly models the post-peak crash, thereby reconciling seemingly contradictory literatures and offering designers a concrete richness range within which gamification remains advantageous.

A second contribution lies in demonstrating that DSE is a boundary condition that recalibrates this SDT–overload curve rather than simply shifting it upward. Prior SCT work has repeatedly shown that efficacy predicts exercise participation in general (Bandura, 1997) and adherence to home-exercise programmers in particular (Picha et al., 2019), but few studies have examined whether efficacy changes how users react to richer—or more cluttered—digital environments. Our zone-specific moderation shows that DSE is inert when cues are scarce, amplifies need-supportive benefits in the optimal range, and buffers need-thwarting costs once overload sets in. This nested pattern extends SCT into the gamified-fitness domain by illustrating that self-efficacy does not merely add a parallel main effect (cf. McAuley et al., 2011); it actively reshapes the curvature of environmental influence. Practically, the finding suggests two complementary levers: raising users’ DSE through guided tutorials can widen the safe richness window, and adaptive interfaces can throttle feature release for low-efficacy profiles to prevent early overload. In sum, the study integrates motivational and cognitive perspectives and pinpoints self-efficacy as the psychological lens that determines whether gamification elements function as fuel or friction.

Additionally, although H2a predicted that DSE would be behaviorally inert when gamified cues were very sparse, the simple-slope test revealed a small but significant negative GFR → adherence slope for low-efficacy students in the extreme left tail (−2 SD to −2.76). At least two complementary explanations may account for this deviation. First, SDT posits that when competence and autonomy cues are not merely absent but noticeably lacking, the setting can actively undermine motivation (Vansteenkiste and Ryan, 2013). Students low in DSE already doubt their ability to navigate digital workouts; encountering an app that supplies almost no guidance or feedback may exacerbate this insecurity, turning indifference into a modest aversion. High-DSE students, by contrast, possess sufficient internal resources to remain unaffected, hence the non-significant slope at the same richness levels. Second, consumer-psychology work shows that minimal feature sets can trigger psychological reactance if users feel deprived of expected functionalities (Brehm, 1981). Because our eligibility screen required at least one app session in the past month, participants arrived with baseline expectations of digital coaching; when those expectations were unmet, low-efficacy users—already uncertain—may have responded with a downward adjustment of adherence intent.

### Practical implications

The findings of this study also provide several practical implications for production managers, university wellness and physical-education administrators. First, the primary audience for the “richness sweet-spot,” self-efficacy scaffolding, and adaptive-throttling tactics is the teams that decide which mechanics ship, how quickly they appear, and how data drives interface adaptation. Progressive-disclosure roadmaps, tutorial pipelines, and real-time feature suppression all live inside the product backlog they control. Second, campus sport departments, student-affairs offices, and e-health units often mandate or recommend specific fitness apps for credit, challenges, or well-being initiatives. Understanding that “more features” can backfire—and knowing how to pair phased feature release with confidence-building workshops—allows them to roll out digital programmers that engage rather than overwhelm students. Third, whether the context is a workplace wellness scheme or a regional public-health partnership, project owners care about KPI retention curves and behavioral outcomes. The study’s thresholds help them set contractual feature limits, require layered onboarding content, and insist on analytics-based throttling clauses when negotiating with third-party app vendors. Fourth, because digital exercise self-efficacy (DSE) amplifies benefits and cushions harms, raising it should be a design priority. Onboarding can begin with a guided “first-workout” tutorial that demonstrates how to navigate menus and interpret feedback. Embedding vicarious success clips—short videos of peers completing workouts—leverages social modeling to boost confidence. Structuring early challenges as incremental, achievable tasks generates early mastery experiences; each success resets the competence baseline upward, making richer feature sets feel less intimidating when they eventually appear.

### Limitations and directions for future research

Despite the theoretical and practical value of our findings, several caveats warrant careful attention and create fertile ground for new studies. First, both the richness perception and the adherence intention were captured in one sitting; although we randomized item blocks and embedded attention checks, common-method inflation and short-term recall bias remain possible. Subsequent work should employ multi-wave or experimental designs that pair subjective ratings with objective app-log data (e.g., session counts, sensor-verified MVPA) to corroborate the S-curve in actual usage behavior. Second, our large but narrow sample of Chinese university students enhances internal validity yet constrains external validity. Exercise norms and technology practices vary across age groups and cultures, which may shift both the “sweet-spot” and “overload” thresholds of gamification richness. We therefore encourage cross-population replications (e.g., working adults and older adults) and cross-cultural tests to examine whether the cubic form and DSE moderation are robust, and how the inflection points relocate with cultural meanings around activity and technology use (Fernandez-Rio and Saiz-González, 2023). Third, feature richness can change weekly as apps update. We treated GFR as a snapshot perception, yet longitudinal data might show that richness trajectories—how quickly features accumulate—matter more than a single level. Growth-curve or time-series modeling could capture how shifts in richness alter motivation over months. Fourth, we centered on self-efficacy, but variables such as personal innovativeness, need for cognition, or tech-related anxiety could also modulate overload effects. Testing multiple moderators simultaneously may reveal user typologies that need distinct design treatments. Fifth, this study measured exercise-adherence intention, which is a validated and useful precursor of behavior. However, intention–behavior gaps are common in physical activity: a recent meta-analysis estimates that nearly half of intenders do not enact their intentions (Feil et al., 2023), and intention strength and stability moderate this link (Conner and Norman, 2022). Future research should triangulate EAI with objective traces (e.g., in-app logs of session counts, streaks, or completed challenges) and device-based activity (accelerometers), to verify whether regime-specific intentions translate into sustained behavior.

## Data Availability

The raw data supporting the conclusions of this article will be made available by the authors, without undue reservation.
